# Understudied and underaddressed: Femicide, an extreme form of violence against women and girls

**DOI:** 10.1371/journal.pmed.1004336

**Published:** 2024-01-18

**Authors:** Chen Reis, Sarah R. Meyer

**Affiliations:** 1 Josef Korbel School of International Studies, University of Denver, Denver, Colorado, United States of America; 2 Institute for Medical Information Processing, Biometry and Epidemiology (IBE), Ludwig-Maximilians University–Munich, Munich, Germany

## Abstract

In this Perspective, Chen Reis and Sarah Meyer discuss the incidence and dynamics of femicide globally and highlight the urgent need for better data and documentation to enact policies aimed at prevention, early detection, and timely intervention in cases of femicide.

## Teaser

Little is known about the prevalence and dynamics of femicide, a persistent form of violence against women and girls, due to challenges associated with its documentation. Research by Abrahams and colleagues comparing rates of femicide in South Africa over 18 years, however, suggests that femicide is preventable.

Femicide—the intentional killing of women and girls because of their gender [1]—is an extreme manifestation of violence against women and girls rooted in misogyny and harmful beliefs and norms. This form of violence, and insufficient responses to it, constitute an undervaluation of the lives of women and girls. Femicide is a public health and human rights issue that is underdocumented, underresearched, and poorly understood especially in lower- and middle-income country settings. In an accompanying research study in *PLOS Medicine*, Abrahams and colleagues report estimates for the prevalence of femicide in 1999, 2009, and 2017, to track femicide rates in South Africa over this 18-year period, finding a reduction in femicide overall and different patterns of change in femicide by category of perpetrator. Building on Abrahams and colleagues‘ previous work on intimate partner femicide [2], this study provides important insight into changes in the nature and prevalence of femicide in South Africa.

Femicide is generally recognized to have 2 subcategories: intimate partner femicide (IPF) committed by former or current intimate partners, which accounts for most cases of femicide, and nonintimate partner femicide (NIPF). Although data are limited, it is estimated that, globally, 89,000 women were killed intentionally in 2022, of which 48,800 were killed by an intimate partner or a family member [3].

Abrahams and colleagues conducted 3 dedicated retrospective survey studies of femicide (which they defined as the intentional killing of women and girls) over 18 years in South Africa using mortuary records [4]. They gathered additional information through interviews with police investigating officers and used weighted cluster designs and hot deck imputation to label cases as IPF or NIPF in cases where the perpetrator was not identified (up to 30% of cases). While there are important limitations associated with this approach and reliance on incomplete records, to the best of our knowledge, the study provides the only South African national-level data of prevalence of IPF and NIPF as well as comparison of variations and patterns by age, racial category, and province. Prior research from the same researchers had compared IPF in 2009 and 1999, finding no difference in rates, whereas NIPF significantly declined from 1999 to 2009 [2].

The overall estimate for IPF in South Africa was 4.9 per 100,000 female population in 2017, which is likely an underestimate [4]. To identify prevalence and document changes in prevalence over time since 1999, the data collection and analysis processes described by Abrahams and colleagues are labor and time intensive and require access to mortuary and police records. This may not be feasible in many lower- and middle-income settings due to a lack of resources and data availability. The authors’ finding that South Africa has one of the highest recorded rates of femicide likely reflects underestimates in other countries due to a lack of reliable data.

Abrahams and colleagues also found an overall decline in femicide over the 18-year period of the study, suggesting that femicide can be prevented, and its prevalence reduced [4]. More research is needed to identify effective measures to reduce femicide. Rates of femicide are driven by, among other factors, endorsement of inequitable gender norms, norms and practices that condone violence against women, and ineffective or inaccessible forms of protection for women at risk of lethal harm. Effective interventions to prevent femicide can include measures to promote equitable gender and social norms, strengthening systems to allow women to report violence and receive protection orders, and reducing access to means of perpetration (i.e., firearms) [5].

Femicide is part of a continuum of violence against women and girls. For example, IPF is often preceded by other forms of intimate partner violence (IPV), including strangulation and rape [6]. As such, risk assessment and policy and programmatic interventions are often built on assumptions that IPV is always a precursor of femicide and that escalation of IPV can culminate in femicide. However, rates of nonlethal violence against women do not always correspond to rates of femicide. In Latin America, high rates of femicide are not mirrored by high rates of self-reported violence against women in survey data [7], and a linear progression from male-perpetrated IPV to femicide cannot be assumed. In an Australian study, more than half of male perpetrators of IPF did not report any physical or sexual violence perpetration in the year prior to committing femicide [8]. While history of IPV can be a precursor to femicide, more research is needed to explore how or whether patterns of previous violence can be predictive of femicide.

Despite the urgency of understanding and preventing femicide globally, there are major gaps in data and evidence synthesis. The most recent global systematic review of intimate partner homicide, conducted a decade ago, found that between a third and half of female homicides globally (depending how gaps in data are addressed) were committed by an intimate partner [9]. Recent research in the European Union indicates that only a few European countries have a centralized database on femicide, and conflicting definitions undermine efforts to understand the nature and extent of femicide [10]. United Nations (UN) agencies note that 133 UN Member States provide homicide data that disaggregates by sex of the victim [11]. However, 4 in 10 female homicides lack contextual information needed to determine whether they are femicides, such as the relationship between perpetrator and victim and any previous harassment or violence, factors relating to the nature of the crime itself (e.g., commission of sexual violence or disposal of body in a public place), and/or victim work in the sex industry, which may be used to categorize homicides as femicide [11]. A large proportion of homicide data globally does not include details on these variables, and, therefore, accurate femicide classification and categorisation is extremely difficult, if not impossible [11]. These gaps in data and issues with data quality result in poorly understood global rates, trends, and predictors of femicide.

The study by Abrahams and colleagues stands out as one of very few comprehensive studies of femicide conducted outside of a high-income country setting. It raises important issues relevant to research, policy, and practice. As noted above, a lack of standardized definition of this form of violence against women and girls and an absence of national and international mechanisms to gather comparable data on femicide confound efforts to identify the magnitude of violence, and changes in prevalence over time. Efforts to address this include the development of a new global framework for measuring gender-related killings of women and girls by the UN Office on Drugs and Crime (UNODC) and UN Women [12]. Abrahams and colleagues’ study indicates the need for both targeted and adequately funded studies to assess prevalence of femicide, particularly in lower- and middle-income countries, as well as investments in data systems, including disaggregation of data by gender, age, and other important characteristics, and recording of perpetrator type and other key factors in routine police and legal records. This will allow for a better understanding of how femicide dynamics and risks differ for different groups of women and girls including adolescents, indigenous women, people of diverse sexual orientation and gender identity, and women and girls involved in sex work.

To reduce the occurrence of femicide, we will need more than detailed documentation and updated reporting of femicide. Policies and laws that target femicide are needed but, even where they exist, they may be inconsistently enforced or implemented due to bias or a lack of political will. Abrahams and colleagues note the importance of work of women’s movements, civil society, and community-based organizations in reducing violence against women and girls, including femicide [4]. While increased documentation and data are key for appropriate prevention and response interventions including early detection, it is also critical to confront structural factors, including gender inequalities, and to reduce violence against women and girls in order to address femicide, a persistent and extreme form of violence against women and girls [5].

## Abbreviations

IPF: intimate partner femicide
IPV: intimate partner violence
NIPF: nonintimate partner femicide
UN: United Nations
UNODC: UN Office on Drugs and Crime
